# The Use of HPV16-E5, EGFR, and pEGFR as Prognostic Biomarkers for Oropharyngeal Cancer Patients

**DOI:** 10.3389/fonc.2018.00589

**Published:** 2018-12-11

**Authors:** Miren Taberna, Montserrat Torres, María Alejo, Marisa Mena, Sara Tous, Sandra Marquez, Miquel A. Pavón, Xavier León, Jacinto García, Marta Guix, Rafael Hijano, Teresa Bonfill, Antón Aguilà, Alicia Lozano, Ricard Mesía, Laia Alemany, Ignacio G. Bravo

**Affiliations:** ^1^Department of Medical Oncology, Catalan Institute of Oncology (ICO), IDIBELL, ONCOBELL, L'Hospitalet de Llobregat, Barcelona, Spain; ^2^Cancer Epidemiology Research Program, Catalan Institute of Oncology (ICO), IDIBELL, L'Hospitalet de Llobregat, Barcelona, Spain; ^3^Department of Medicine, University of Barcelona, Barcelona, Spain; ^4^Cancer Epidemiology Research Program, Infections and Cancer Laboratory, Catalan Institute of Oncology-IDIBELL, L'Hospitalet de Llobregat, Barcelona, Spain; ^5^Department of Pathology, Hospital General de L'Hospitalet, L'Hospitalet de Llobregat, Barcelona, Spain; ^6^Centro de Investigación Biomédica en Red de Cáncer (CIBERONC), Madrid, Spain; ^7^Otorhinolaryngology Department, Hospital de Sant Pau, Barcelona, Spain; ^8^Centro de Investigación Biomédica en Red de Bioingeniería, Biomateriales y Nanomedicina (CIBER-BBN), Madrid, Spain; ^9^Cancer Research Program, IMIM, Hospital del Mar, Barcelona, Spain; ^10^Department of Medical Oncology, Hospital del Mar, Barcelona, Spain; ^11^Department of Otorhinolaryngology, Hospital del Mar, Barcelona, Spain; ^12^Department of Medical Oncology, Hospital Universitari Parc Taulí, Sabadell, Barcelona, Spain; ^13^Department of Otorhinolaryngology, Hospital Universitari Parc Taulí, Sabadell, Barcelona, Spain; ^14^Department of Radiation Oncology, Catalan Institute of Oncology (ICO), IDIBELL, L'Hospitalet de Llobregat, Barcelona, Spain; ^15^Department of Medical Oncology, Catalan Institute of Oncology (ICO), Hospital Can Ruti, Badalona, Spain; ^16^Centro de Investigación Biomédica en Red: Epidemiología y Salud Pública (CIBERESP), Instituto de Salud Carlos III, Madrid, Spain; ^17^French National Center for Scientific Research (CNRS), Laboratory MIVEGEC (CNRS IRD Uni Montp), Montpellier, France

**Keywords:** head and neck cancer, oropharyngeal cancer, Human Papillomavirus, HPV, EGFR, pEGFR, HPV16, HPV16-*E5*

## Abstract

**Background:** Anti-epidermal-growth-factor-receptor (EGFR) therapies in combination with radiotherapy are being studied on de-escalation clinical trials for HPV-related oropharyngeal cancer (OPC) patients. The HPV16-E5 oncoprotein increases recycling of activated EGFR to the cell surface, enhancing factor signal transduction. Our aim was to evaluate viral HPV16-*E5* oncogene expression as well as EGFR and phosphorylated-EGFR (pEGFR), protein levels as biomarkers for clinical outcome in a retrospective cohort of OPC patients.

**Methods:** Formalin-fixed-paraffin-embedded OPCs were collected from 1990 to 2013. OPC samples containing HPV-DNA were subject to viral E6^*^I mRNA detection and p16^INK4a^ immunohistochemistry (IHC). HPV16-positive cases were evaluated for HPV16-*E5* (RT-PCR) and EGFR/pEGFR (IHC). A stratified and matched random sample of HPV-negative samples was used as control and evaluated for EGFR/pEGFR. Overall survival (OS) and disease free survival (DFS) estimates were assessed for locally advanced OPC patients (stage III, IVa,b 7th edition).

**Results:** Among 788 OPC patient samples, 53 were double positive for HPV16-DNA/p16^INK4a^. HPV16-*E5* expression was found in 41 of 53 samples (77.4%). EGFR expression was observed in 37.7 *vs* 70.8% of HPV16-positive *vs* HPV-negative samples, respectively; (adjusted OR = 0.15) 5% CI = 0.04–0.56]). Expression of pEGFR followed an inverse pattern with 39.6 and 24.9% detection in HPV16-positive and HPV-negative samples; (adjusted OR = 1.58 [95% CI = 0.48–5.17]). Within HPV16-positive cases, no association between HPV16-*E5*/EGFR nor pEGFR was observed. With a median follow-up of 39.36 months (min = 0.03 – max = 272.07), the combination of HPV status and EGFR or pEGFR expression were predictors of better OS (*p* < 0.001, for both) and DFS (*p* < 0.001 for EGFR and *p* = 0.003 for pEGFR).

**Conclusions:** HPV16-*E5* is highly expressed on HPV16-positive OPCs. Interestingly, HPV16-positive cases expressed significantly more pEGFR while HPV-negative cases expressed more EGFR. The combinations of HPV status and EGFR or pEGFR may be useful biomarkers for evaluating prognosis outcome in OPC patients.

## Introduction

Chronic infection by oncogenic Human Papillomaviruses (HPVs) is the principal cause of the increasing incidence rates of oropharyngeal carcinoma (OPC) (1).

Viral transformation concurs with a lower mutational burden, resulting in better prognosis for patients with HPV-related OPCs (2). Given the high morbidity associated with current treatments (3), the scientific community is seeking de-escalation protocols aiming at maintaining the cure rates while reducing toxicity. Therapies targeting the Epidermal Growth Factor Receptor (EGFR) have been proposed as de-escalation strategies in HPV-related OPC patients, as an alternative to cisplatin when given concurrently with radiation therapy, attempting to reduce cisplatin side-effects (4, 5). Other de-escalation therapies include reduction of radiation dose given in combination with chemotherapy as primary treatment, reduced dose of radiotherapy with or without cisplatin or reduction of adjuvant chemo-radiotherapy dose following primary treatment with surgery (4, 5). EFGR is overexpressed in 90% of head and neck squamous cell carcinomas (HNSCC) (6). Nevertheless, there is limited evidence supporting the role of anti-EGFR therapy in HPV-related cancers, as alteration in EGFR levels in OPCs does not correlate with HPV status (7, 8), and clinical data remain controversial (7). Ligand-dependent EGFR activation leads to auto-phosphorylation, and the activated form pEGFR is the responsible molecular species that triggers the downstream signaling cascade, eventually stimulating cell division (9).

Association with HPV infection, EGFR expression level, and history of tobacco exposure are well-characterized prognosis factors in HNSCC: HPV-related transformation is associated with improved outcome (2); EGFR overexpression is associated with poor prognosis (10); and tobacco may contribute to increased EGFR expression through increased local hypoxia in tumor tissue (6). Along this line, pEGFR has recently been described as a prognosis biomarker related to poorer outcomes in HNSCC (9, 11).

The direct effects of the E6 and E7 HPVs oncoproteins at inactivating respectively p53 and retinoblastoma tumor suppressor proteins and thereby disturbing cell cycle regulation are well known as the hallmarks of HPVs-related transformation (5, 12). However, the functions of the third viral oncoprotein, E5, remain poorly understood (13). Expression of HPV16-*E5* results in diverse effects, modifying the membrane chemistry and influencing a variety of pathways involved in growth factor-dependent cell proliferation, immune recognition, and altered response to extrinsic and intrinsic pro-apoptotic signals (14). In the present work we have focused on the ability of HPV16-E5 at inhibiting endosomal acidification, thereby promoting recycling of activated pEGFR to the cell surface (15, 16). Previous studies have shown that HPV16-*E5* encoding transcripts are variably expressed in HPV-related HNSCC, and that *E5* gene expression correlates with EGFR overexpression in HPV-related OPC (13). Current data on the differential response of OPCs to anti-EGFR therapies as a function of the HPV status are still inconsistent and importantly, 20% of HPV-related OPSCC patients fail to treatment (2). Here we have analyzed for the first time HPV16-*E5* expression, EGFR and pEGFR cellular levels, as well as smoking status in a retrospective cohort of OPC patients. In an era where anti-EGFR therapies are being studied on de-escalation clinical trials we aimed at evaluating the prognostic and predictive values of these biomarkers and their differential connection with clinical outcome.

## Materials and Methods

### Study Population

We carried out a large retrospective cohort study (17), to assess the prognostic and predictive value of HPV-related carcinogenic biomarkers, on consecutively selected OPC cases from four different hospitals from Catalonia (Catalan Institute of Oncology-ICO-Hospital Universitari de Bellvitge; Hospital de Sant Pau, Hospital del Mar and Hospital Parc Taulí) from 1990 to 2013. The current study is nested within this larger cohort and was designed to evaluate the variation in EGFR and pEGFR cellular levels, as well as in HPV16-*E5* expression, in the context of clinical outcomes. All HPV16-positive cases [as defined by double positivity for HPV-DNA and p16^INK4a^, (*n* = 53, 6.7%) were eligible to participate. A random set of HPV-negative samples (*n* = 174) matched for tobacco use were chosen as control group [random sample 1:3 for smokers (< 20 cigarettes a day or ≥20 cigarettes a day) and 1:1 for non-smokers]. The 1–3 matching for non-smokers was not possible due to limited sample size in this group.

Cases were confirmed by anatomopathology and metastatic patients were discarded (7th TNM edition). Demographic and clinical information was extracted from the corresponding clinical reports. Overall survival (OS) and disease free survival (DFS) were assessed up to five years after diagnosis. All methods were carried out in accordance with relevant guidelines and regulations. The protocol was approved by the Institutional Review Broad and ethical committee of each participating hospital (PR323/14).

### Laboratory Analysis

Biomarker assessment was centralized at the Catalan Institute of Oncology (Barcelona, Spain) in collaboration with Hospital General de L'Hospitalet (Barcelona, Spain) and the German Cancer Research Center (DKFZ, Heidelberg, Germany), using OPC formalin-fixed paraffin-embedded (FFPE) samples. The detailed methods for immunohistochemistry (IHC), HPV-DNA detection, genotyping, and HPV16-E6^*^I mRNA assessment have been reported elsewhere (18). Briefly, hematoxylin and eosin stained slides were used to confirm presence and estimate the proportion of invasive SCC in the specimen as well as to classify histopathological features. HPV-DNA detection and genotyping was performed using SPF10-DEIA-LiPA25 version 1 system (Labo Biomedical Products, Rijswijk, Netherlands). All HPV-DNA positive samples underwent RNA extraction and HPV-E6^*^I mRNA detection, as previously reported (19). All HPV16-positive cases were further evaluated for HPV16-*E5* mRNA by reverse transcription quantitative PCR (RT-qPCR). The RT-qPCR was designed to generate a 94 base pairs-long amplicon within the HPV16-*E5* gene, as previously described (13). BRYT-Green® based RT-qPCR was performed using Light Cycler® 96 Real-Time PCR System (Roche Applied Science, USA) in a final volume of 20 μL reaction using GoTaq^®^ 1-Step RT-qPCR System (Promega, USA), 0.1 μM of each forward and reverse primer and 3 μL of RNA sample. The thermal cycler conditions programmed were 15 min at 37°C and 10 min at 95°C, followed by 45 cycles of 10 s at 95°C, 10 s at 64°C and 10 s at 72°C. Following amplification, melting curve analysis was performed to assess the nature of the PCR product using a melting program with an increase of 2.2°C/s from 65 to 97°C. RNA from cell lines 93-VU-147T (originated from a floor of mouth squamous cell carcinoma, kindly provided by J. Dorsman) and SCC-9 ATCC® CRL-1629™ (human tongue squamous cell carcinoma) were used as HPV16-positive and HPV-negative controls, respectively. In all samples that tested negative for the presence of HPV16-*E5* mRNA, the presence of the target HPV16-*E5* DNA was confirmed by PCR using the same amplification primers.

### Histopathological Evaluation

We used Roche mtm Laboratories AG IHC (Heidelberg) for p16^INK4a^ determination. p16^INK4a^ IHC was considered positive when the pattern showed a strong and diffuse nuclear and cytoplasmic staining in at least 70% of the cancer tissue (20). A case was consider HPV-related if both HPV-DNA PCR and p16^INK4a^ IHC determination were positive, following the recommendation from a previous study from our group (17) and from a recent meta-analysis (21). Of note, all samples testing double positive for HPV16-DNA and p16^INK4a^ were also positive for HPV E6^*^I mRNA.

**Figure 1:**
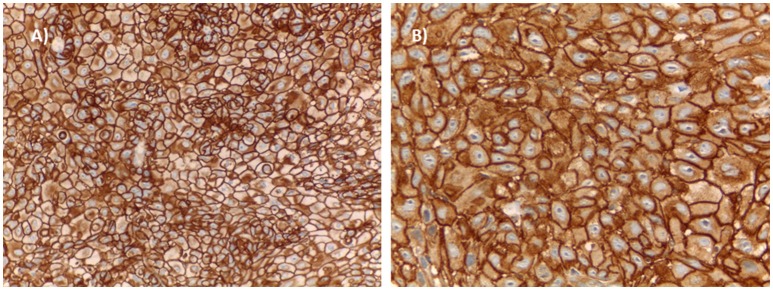
Histopathological examples of OPC with EGFR **(A)** and pEGFR **(B)** expression.

All HPV16/p16^INK4a^-positive samples as well as the stratified random sample of HPV-negative cases were tested for IHC staining for EGFR [anti-EGFR (3C6), Roche; pre-diluted] and pEGFR [EP774Y, Abcam, (Tyr1068); dilution 1/300]. Membranous and sub-membranous cytoplasmic staining was considered acceptable for either marker, according to manufacturers' data. EGFR protein and pEGFR expression levels were evaluated semi-quantitatively and classified as follows: Score 0: no staining or membrane staining in < 10% of tumor cells; Score 1+: faint membrane staining in >10% of tumor cells; Score 2+: weak or moderate complete membrane staining in >10% of tumor cells and Score 3+: strong, complete membrane staining in >10% of tumor cells. Scores of 0 and 1+ were considered negative for either EGFR or pEGR expression while Scores 2+ and 3+ were considered positive. Representative images for EGFR and pEGFR positive samples are displayed on Figure 1.

### Statistical Analyses

Conditional logistic models accounting for matching on tobacco use were used to assess the association between HPV16-positive OPCs and EGFR or pEGFR expression. Crude and adjusted odds ratios for age, sex, alcohol consumption site, and treatment, and their 95% confidence intervals were estimated. Histological variables were not considered in multivariate analyses, as previously described (22). Differences in baseline characteristics between HPV16-positive and HPV-negative cases were compared using the paired *t*-test for continuous variables and the Fisher's exact test for categorical variables. Agreement between EGFR and pEGFR stratified by HPV status was assessed through Kappa index and the distribution of discordant responses from EGFR and pEGFR by using McNemar's test. Those were also used to assess the agreement/discordant cells between HPV16-*E5* and EGFR or pEGFR expression in HPV16-positive OPC cases. For survival analysis only patients with locally advanced disease (stage III, IVa,b 7th edition) were included. Survival time was calculated from the date of histological diagnosis to either time of death (overall survival, OS) or to time of cancer recurrence (disease-free survival, DFS). The cumulative probability of survival past five years was estimated by Kaplan–Meier analysis. Survival curves were compared by means of the log-rank test. All *p*-values obtained were corrected for multiple testing by using the Benjamini-Hochberg procedure with alpha = 0.05 significance level. Multivariate Cox's proportional hazards models were performed under three scenarios to explore whether HPV16-positivity, EGFR and pEGFR expression alone or any combination between them could have a value as prognostic variables, after accounting for other confounding factors (see Table 3 foot note). Hazard ratios were calculated for death and recurrence for OPC patients. Statistical analyses were performed with STATA SE version 15.0 and R version 3.3.2.

## Results

**Figure 2:**
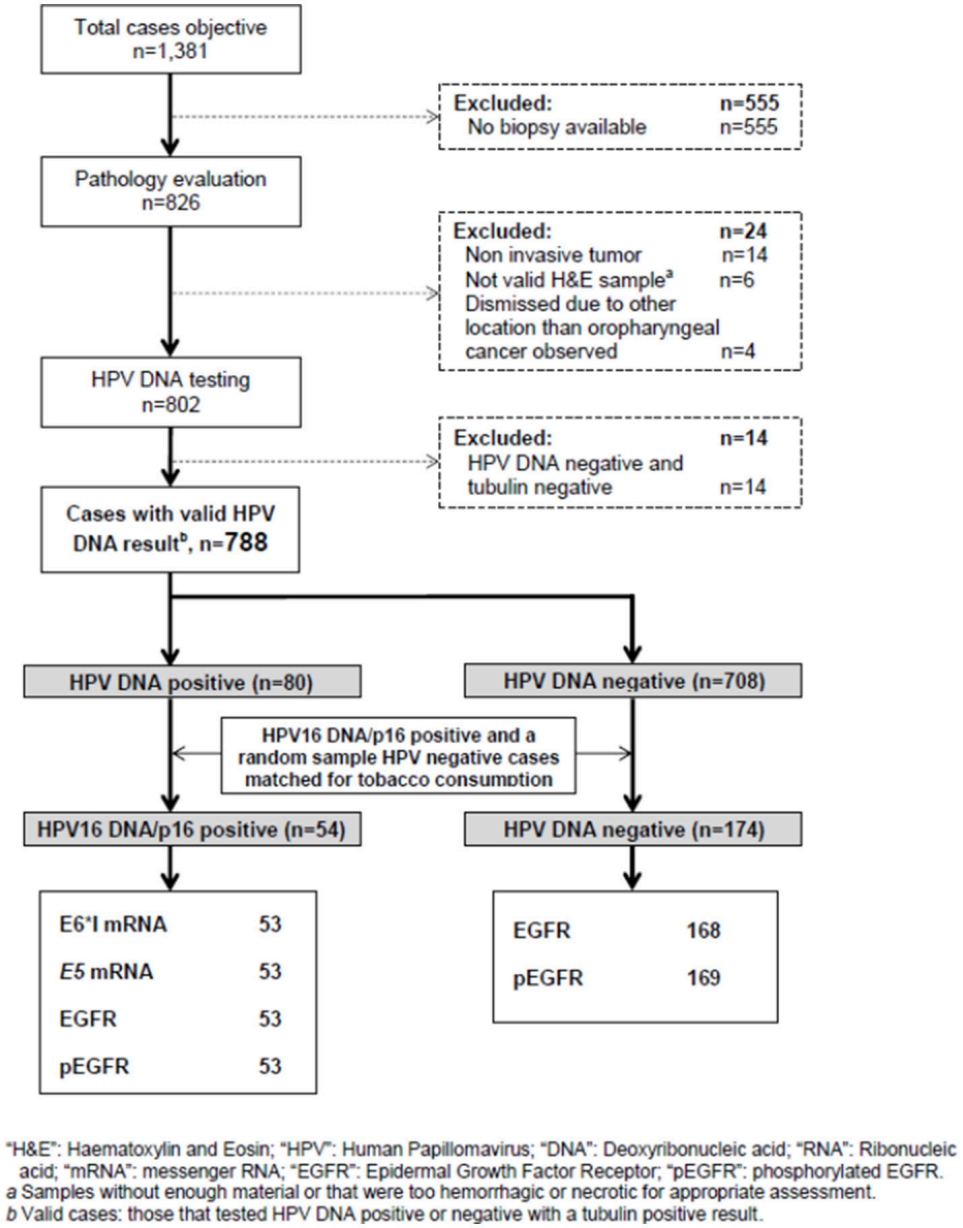
Samples disposition and testing for HPV status and all biomarkers.

Figure 2 describes the workflow of the OPC targeted cases, samples collected, processed and tested. Among the 1,381 OPC patients targeted, a total of 788 samples yielded a valid DNA result and were finally included in the analysis. HPV-DNA positivity was found in 80 (10.2%) samples and HPV16 was the most frequent genotype, present in 67 cases (83.7% among HPV-DNA positive cases). The number of *bona-fide* HPV16-positive cases when considering double positivity for HPV16-DNA and p16^INK4a^ IHC was 53 (67.5% among HPV-DNA positive cases). Hereinafter such double positive cases will be referred to as HPV16-positive cases. A random sample of 174 HPV-negative cases matched for tobacco use was also included in the analysis as a control group. A demographical and clinical descriptive of the OPC patients by HPV status is summarized in Supplementary Table S1. HPV16-positive patients were significantly more likely to be female, non-drinkers, to have an undifferentiated tumor or to have a primary tonsil tumor. The demographic and clinical characteristics associated with HPV16-*E5* expression, EGFR and pEGFR levels, stratified by HPV status, as well as the crude Odds Ratio (OR) measures of association are shown in Supplementary Table S2. The percentage of HPV16-positive cases expressing also HPV16-E5 was 77.4% (41/53). Since HPV16 integration often concurs with *E5* ablation, all samples that tested negative for the presence of HPV16-*E5* mRNA (12/53) were further tested for HPV16-*E5* DNA, and 58.3% (7/12) were positive. We confirmed that these seven HPV16-*E5* mRNA-negative/HPV16-*E5* DNA-positive samples were also positive for HPV-E6^*^I mRNA, thus excluding the possibility of a false negative for mRNA presence by mRNA degradation.

We examined differential EGFR and pEGFR levels between HPV16-positive OPCs and tobacco smoking-matched HPV-negative OPCs and calculated the corresponding ORs (Table 1). EGFR expression levels were significantly lower in HPV16-positive OPCs than in HPV-negative: 37.7% (20/53) *vs* 70.8% (119/168), respectively (OR = 0.15 [95% Confidence Interval (CI): 0.04–0.56]). There were no differences in pEGFR expression levels between HPV16-positive and HPV-negative OPCs: 39.6% (21/53) *vs* 24.9% (42/169), respectively (OR = 1.58 [95%CI: 0.48–5.17]). Furthermore, as a sensitivity analysis, we reanalyzed the data using unconditional logistic regression obtaining similar results: for EGFR expression levels, OR = 0.22 [95%CI: 0.12–0.52]; for pEGFR, OR = 2.02 [95%CI: 0.87–4.7]. Table 2a shows the concordance between EGFR and pEGFR expression by HPV status. Among HPV-negative OPC patients we observed an unequal distribution of discordant samples, with an unbalance toward higher proportion of EGFR-positive/pEGFR-negative cases, not observed in HPV16-positive cases (McNemar's test *p* < 0.001 and 1.0, respectively). Within HPV16-positive cases, no agreement was found between HPV16-*E5* expression and EGFR or pEGFR levels (Kappa = −0.03 and Kappa = −0.08, respectively; Table 2b).

**Table 1:** Association of differential EGFR and pEGFR levels between HPV16-positive OPCs and tobacco smoking-matched HPV-negative OPCs.

**Biomarker**	**HPV status**		OR [95% CI]*	OR [95%CI]**
	**HPV negative**	**HPV16 positive**	
	**No/Total (%)**	**No/Total (%)**	
**EGFR EXPRESSION**				
Negative	49/168 (29.2)	33/53 (62.3)	Ref.
Positive	119/168 (70.8)	20/53 (37.7)	0.15 [0.04–0.56]
**pEGFR EXPRESSION**				
Negative	127/169 (75.1)	32/53 (60.4)		Ref.
Positive	42/169 (24.9)	21/53 (39.6)		1.58 [0.48–5.17]

* *Adjusted OR by EGFR expression, age, sex, alcohol consumption, and site. Cases matched by tobacco use (see details in Methods section)*.

** *Adjusted OR by pEGFR expression, age, sex, alcohol consumption, and site. Cases matched by tobacco use (see details in Methods section).EGFR, Epidermal Growth Factor Receptor; pEGFR, phosphorylated EGFR; HPV, Human Papillomavirus; OPC, Oropharyngeal carcinoma; OR, Odds Ratio; 95%CI, 95% Confidence Interval. HPV16 positive cases are also positive for p16^INK4a^*.

**Table 2a:** Concordance between EGFR and pEGFR expression by HPV status.

**EGFR expression**	**pEGFR expression**		**Kappa [95%CI]**	**McNemar's test *P*-value**
	**Negative**	**Positive**	
	***N* (%)**	***N* (%)**	
**HPV16 POSITIVE**				
Negative	23 (43.4)	10 (18.9)	
Positive	9 (17.0)	11 (20.8)	0.24 [−0.02 to 0.51]	1.000
**HPV/DNA NEGATIVE**				
Negative	38 (22.6)	11 (6.5)	
Positive	88 (52.4)	31 (18.5)	0.03 [−0.07 to 0.12]	< 0.001

*HPV, Human Papillomavirus; EGFR, Epidermal Growth Factor Receptor; pEGFR, phosphorylated EGFR. DNA, Deoxyribonucleic acid; HPV16 positive cases are also positive for p16^INK4a^. Kappa index: values < 0 as indicating no agreement and 0–0.20 as slight, 0.21–0.40 as fair, 0.41–0.60 as moderate, 0.61–0.80 as substantial, and 0.81–1 as almost perfect agreement*.

**Table 2b:** Concordance between HPV16-*E5* expression and EGFR or pEGFR expression in HPV16 positive OPC cases.

***E5* mRNA status**	**EGFR expression**		**Kappa [95%CI]**	**McNemar's test *P*-value**
	**Negative**	**Positive**	
	***N* (%)**	***N* (%)**	
Negative	7 (13.2)	5 (9.4)	
Positive	26 (49.1)	15 (28.3)	−0.03 [−0.23 to 0.16]	< 0.001
***E5*** **mRNA status**	**pEGFR expression**		**Kappa [95%CI]**	**McNemar's test** ***P*****-value**
	**Negative**	**Positive**	
	***N*** **(%)**	***N*** **(%)**		
Negative	6 (11.3)	6 (11.3)	
Positive	26 (49.1)	15 (28.3)	−0.08 [−0.29 to 0.12]	< 0.001

*HPV, Human Papillomavirus; EGFR, Epidermal Growth Factor Receptor; pEGFR, phosphorylated EGFR. HPV16 positive cases are also positive for p16^INK4a^. Kappa index: values < 0 as indicating no agreement and 0–0.20 as slight, 0.21–0.40 as fair, 0.41–0.60 as moderate, 0.61–0.80 as substantial, and 0.81–1 as almost perfect agreement*.

**Figure 3:**
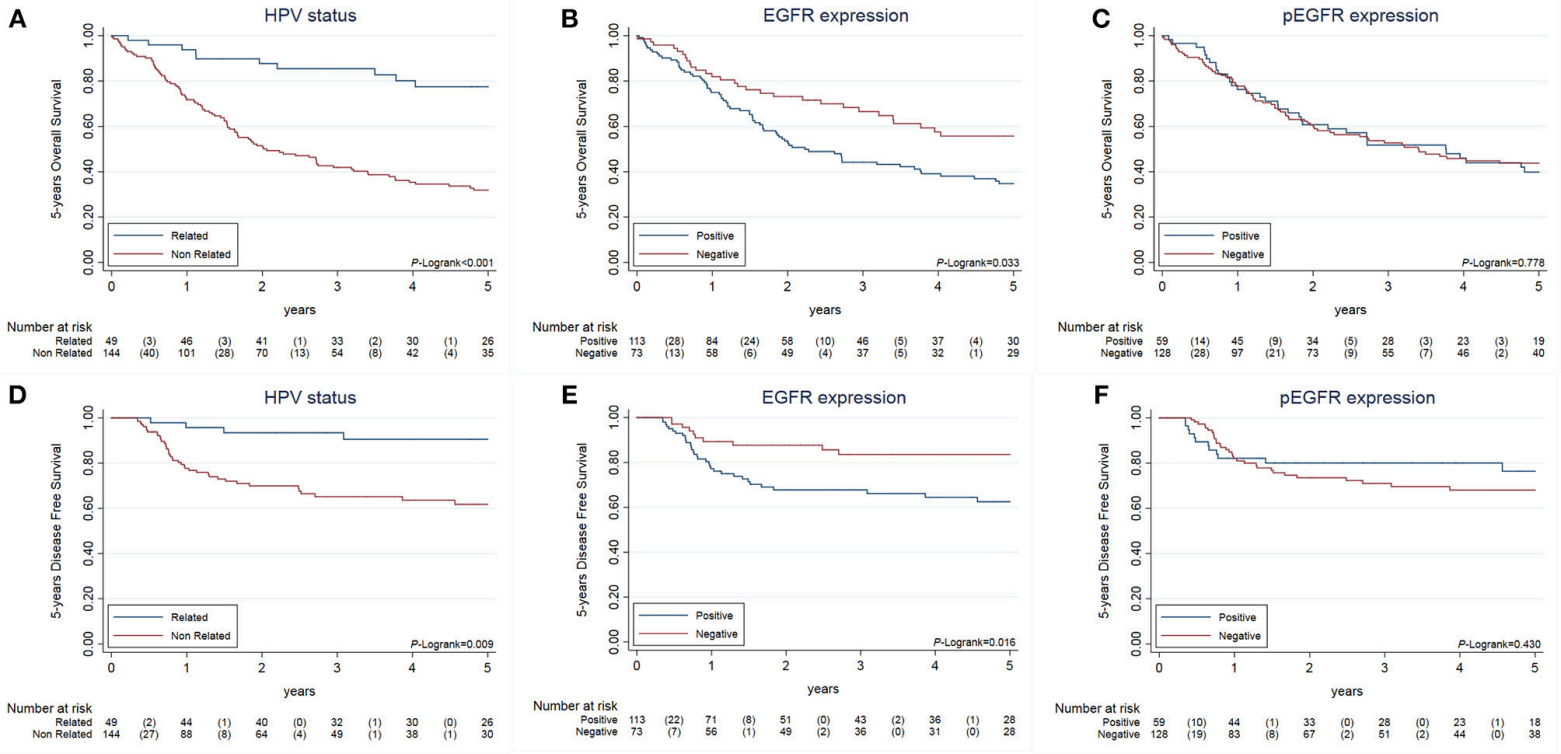
Overall survival and disease free survival of locally-advance OPC patients based on Kaplan-Meier curves stratified by biomarkers. Legend: Data on 5 years Overall Survival **(A–C)** and 5 years disease free survival **(D–F)** according to three different biomarkers: HPV16 **(A,D)**, EGFR **(B,E)**, and pEGFR **(C,F)**. **(A,D)** Show OS and DFS Kaplan-Meier curve for HPV16-related and HPV negative samples. **(B,E)** Show OS and DFS Kaplan-Meier curve for EGFR-positive and EGFR-negative samples. **(C,F)** Show OS and DFS Kaplan-Meier curve for pEGFR-positive and pEGFR-negative samples.

With a median follow-up of 39.36 months (min = 0.03 – max = 272.07), survival analyses were performed. We examined the OS and DFS for OPC patients based on Kaplan-Meier curves stratified by different biomarkers. We first evaluated the role of each biomarker alone (Figure 3). HPV16-positive cases demonstrated better OS (a) Log-rank *p* < 0.001 and DFS (d) Log-rank *p* = 0.009. In the same line, EGFR-negative cases demonstrated better OS (b) Log-rank *p* = 0.033 and DFS (e) Log-rank *p* = 0.016. We did not find differences related to pEGFR detection alone neither in OS (c) Log-rank *p* = 0.778 nor in DFS (f) Log-rank *p* = 0.430.

**Figure 4:**
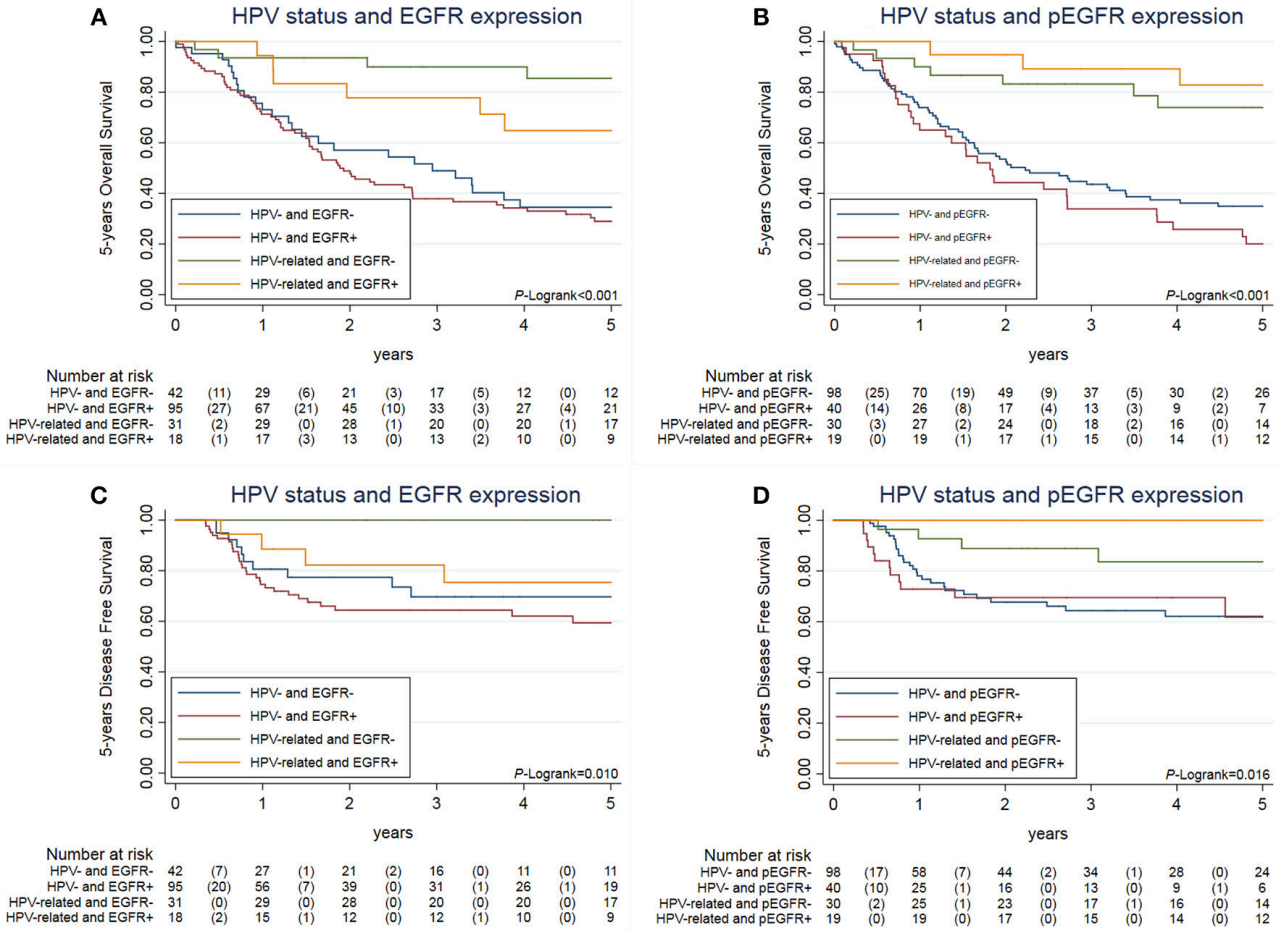
Overall survival and disease free survival of of locally-advance OPC patients stratified by combination of HPV16 status and EGFR or pEGFR. Legend: Data on 5 years Overall Survival **(A,B)** and 5 years disease free survival **(C,D)** according to the combination of HPV16 status and EGFR or pEGFR. **(A,C)** Show OS and DFS Kaplan-Meier curve according to HPV16 status and EGFR expression. **(B,D)** Show OS and DFS Kaplan-Meier curve according to HPV16 status and pEGFR expression. The *H0* assumed that all groups have equal OS and DFS.

We further evaluated the differences in OS and DFS as a function of the combination of HPV status and EGFR expression (Figures 4A,C) and of HPV status and pEGFR levels (Figures 4B,D). The analyses showed that stratification after HPV positive/negative and EGFR or pEGFR positive/negative resulted in differences in both OS and DFS (Log-rank *p*-values indicated in the corresponding figure).

**Figure 5:**
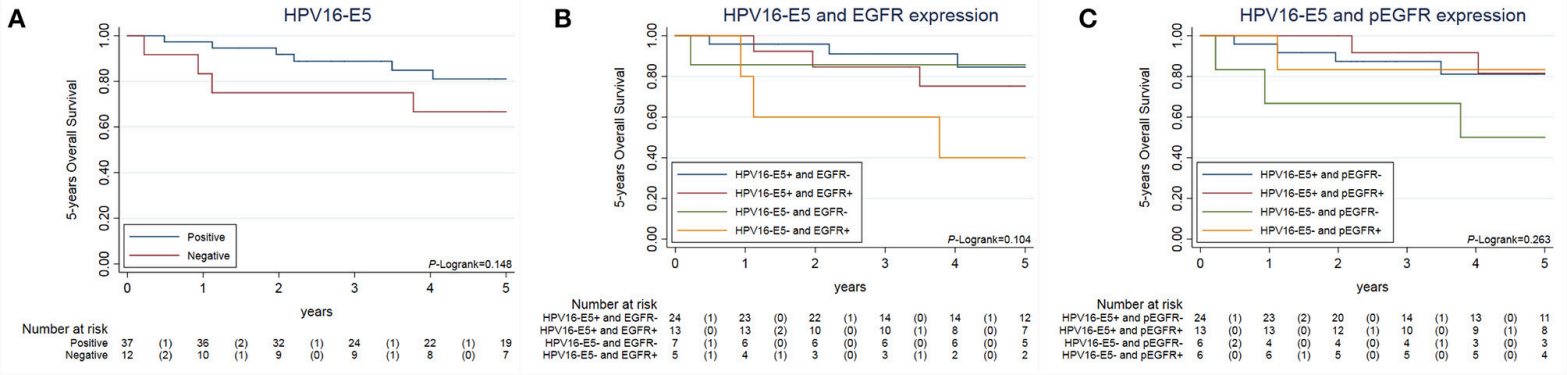
Overall survival of locally-advanced HPV16-related patients stratified by HPV16-E5 alone and in combination with EGFR and pEGFR. Legend: Data on 5 years Overall Survival for HPV16-related patients, stratified by HPV16-E5 **(A)** the combination of HPV16-E5 and EGFR **(B)** and the combination of HPV16-E5 and pEGFR **(C)**. The *H0* assumed that all groups have equal OS.

Among HPV16-positive cases, when analyzing the impact on survival of HPV16-*E5* detection, patients with OPC expressing *E5* displayed better OS than *E5*-negative ones, but this difference was not significant (Log-rank *p* = 0.148; Figure 5A). Finally, evaluation of differences in OS and DFS as a function of the combination of HPV16-*E5* and EGFR (Figure 5B) or pEGFR (Figure 5C) expression did not show differences between groups (Log-rank *p* = 0.104 and Log-rank *p* = 0.263, respectively).

Hazard ratios (HR) for death and recurrence for OPC patients are presented in Table 3, stratifying by HPV16-positivity, EGFR, and pEGFR expression and their combinations. When all three biomarkers were considered separately and after adjusting for confounding covariates, differences in OS and DFS among OPC patients remained statistically significant for HPV16-positive compared to negative and for EGFR positive *vs* negative regarding adjusted HR for DFS. When combining HPV16 status and EGFR expression, HPV16-positive/EGFR-negative samples demonstrated better OS than any other combination (HR = 0.13 [95%CI: 0.05–0.36]). For HPV16 status and pEGFR expression, all combinations showed better OS compared to patients HPV-negative/pEGFR-positive (*p* < 0.001).

**Table 3:** Adjusted and unadjusted Hazard Ratio and 95% confidence interval for Cox proportional hazard models predicting 5-years all-cause mortality and 5-years recurrence in locally-advanced oropharyngeal cancer patients.

**All cases**	**5-years overall survival**				**5-years disease free survival**			
	**Crude HR [95%CI]**	***P*-value**	**Adjusted HR [95%CI]**	***P*-value**	**Crude HR [95%CI]**	***P*-value**	**Adjusted HR [95%CI]**	***P*-value**
**SCENARIO 1: MARKERS CONSIDERED SEPARATELY**								
**EGFR expression**
Negative	Ref.		Ref.		Ref.		Ref.
Positive	**1.69 [1.09–2.62]**	**0.015**	1.36 [0.87–2.14]	0.174	**2.43 [1.19–4.96]**	**0.009**	**2.25 [1.08–4.66]**	**0.021**
**pEGFR expression**
Negative	Ref.		Ref.		Ref.		Ref.
Positive	1.06 [0.70–1.61]	0.779	1.02 [0.66–1.57]	0.921	0.77 [0.39–1.49]	0.423	0.95 [0.48–1.91]	0.893
**HPV16 status**
Negative	Ref.		Ref.		Ref.		Ref.
Positive	**0.22 [0.11–0.43]**	**<0.001**	**0.24 [0.12–0.48]**	**<0.001**	**0.20 [0.07–0.59]**	**<0.001**	**0.19 [0.07–0.57]**	**<0.001**
**SCENARIO 2: COMBINED HPV16 STATUS AND EGFR EXPRESSION**								
HPV16 positive and EGFR positive	**0.34 [0.14–0.82]**		**0.38 [0.15–0.95]**		0.53 [0.17–1.59]		0.42 [0.13–1.31]
HPV16 positive and EGFR negative	**0.13 [0.05–0.36]**		**0.17 [0.06-0.48]**		*		*
HPV16 negative and EGFR positive	Ref.		Ref.		Ref.		Ref.
HPV16 negative and EGFR negative	0.84 [0.53–1.34]	**<0.001**	1.07 [0.65–1.77]	**<0.001**	0.71 [0.34–1.47]	**<0.001**	0.95 [0.43–2.10]	**<0.001**
**SCENARIO 3: COMBINED hpv16 STATUS AND pEGFR EXPRESSION**								
HPV16 positive and pEGFR positive	**0.13 [0.04-0.42]**		**0.14 [0.04–0.47]**		*		*
HPV16 positive and pEGFR negative	**0.21 [0.09–0.49]**		**0.25 [0.10–0.60]**		0.34 [0.10–1.09]		**0.26 [0.08–0.90]**
HPV16 negative and pEGFR positive	Ref.		Ref.		Ref.		Ref.
HPV16 negative and pEGFR negative	0.77 [0.50–1.19]	**<0.001**	0.79 [0.50–1.26]	**<0.001**	0.95 [0.48–1.89]	**<0.001**	0.69 [0.33–1.42]	**0.001**

*P-value: Log-likelihood ratio test P-value*.

* *Combinations that do not contain individuals with events of recurrence*.

*Cases matched by tobacco use (see details in Methods section). In all cases, values in bold correspond show statistical significant different from the null hypothesis of the test*.

***Overall survival**. Variables included in the adjusted models: Scenario 1: age, gender, and treatment, and EGFR expression or pEGFR expression or HPV16 status; Scenario 2: combined HPV16 status and EGFR expression and age, gender, and treatment; Scenario 3: combined HPV16 status and pEGFR expression and age, gender, and treatment*.

***Disease-free survival**. Variables included in the adjusted models: Scenario 1: age, gender, and period of diagnosis, and EGFR expression or pEGFR expression or HPV16 status (without period of diagnosis); Scenario 2: combined HPV16 status and EGFR expression, age, gender, period of diagnosis and subsite; Scenario 3: combined HPV16 status and pEGFR expression, age, gender, and period of diagnosis*.

## Discussion

Viral etiology (*i.e*., HPV-related status) is already well established as an independent predictor of improved OS and treatment responsiveness in OPCs (2, 5). Notwithstanding, not all patients with HPV-related OPCs respond equally to treatment nor have the same prognosis. With this study we have aimed at identifying additional biomarkers beyond OPC-relatedness to better classify HPV-related OPC patients, in an era where developing de-escalation clinical trials is a translational research priority.

We have adhered to a strict definition of viral etiology of OPC samples: an OPC was defined as HPV16-positive if it tested positive for the presence of viral HPV16-DNA and also displayed p16^INK4a^ high expression—a cellular surrogate marker for oncoviral activity (17). In our series, HPV16 was the most frequently detected viral genotype, accounting for 83% among HPV-DNA positive OPC samples, matching previous descriptions for the oropharynx (18, 23). Among HPV16-positive samples, 77.4% expressed HPV16-*E5*, in agreement with Um and coworkers, who described variable *E5* expression in HPV16-positive OPCs (13).

During chronic infection and malignization, viral DNA can integrate into the cellular DNA (24, 25). Usually, this integration event concurs with the loss of particular stretches of the viral genome, often spanning the *E2* and the *E5* genes. For this reason, in all twelve HPV16-*E5* mRNA negative samples we assessed the presence of the corresponding *E5* target DNA, as well as the expression of the HPV16-E6^*^I mRNA. All twelve samples were positive for *E6* expression, further confirming viral transcriptional activity and thus active viral gene expression. Seven of them (58%) were HPV16-*E5* DNA-positive, suggesting that the *E5* oncogene was present but its expression had been shut down, either because of upstream disruption of the viral genome or because of epigenetic silencing (26). In five of the HPV16-*E5* mRNA negative samples we could not detect the *E5* DNA, suggesting that it had been probably deleted during an integration event.

The E5 oncoprotein enhances the transforming activity of E6 and E7 (13). We therefore aimed at studying whether HPV16-*E5* expression could be an additional prognostic biomarker for HPV16-positive OPC patients. In our series, detection of HPV16-*E5* mRNA suggested an improved OS, but this trend was not significant, possibly due to the limited size of our cohort (*N* = 53). Our results thus are concordant with but cannot confirm previous studies reporting an improved DFS, but not OS for patients with cancers displaying high HPV16-*E5* expression levels (13).

EGFR is frequently reported to be overexpressed in HNSCC, and such overexpression correlates with poor prognosis: almost 80% of HNSCC studies demonstrate decreased OS and DFS for patients with cancers overexpressing EGFR (27). Mutations in the *EGFR* gene, such as gene amplification or constitutive activation are common among non HPV-related HNSCCs (8, 28, 29). Results from our series at the protein level are consistent with this trend, as HPV-negative samples expressed more often EGFR than HPV16-positive ones (71 *vs* 38%). Results from the report by Kumar and coworkers. assessing EGFR expression and HPV-relatedness status in OPCs (30) are consistent with those communicated here, but in their case EGFR expression was higher for both groups (93 *vs* 78%, respectively). This large variation, most notable among HPV-positive cases, can stem from (i) the different evaluation criteria used for assessing EGFR expression (their three-tier *vs* our two-tier classification scheme), (ii) the HPV-relatedness definition use (more strict in our case), or (iii) may simply be related to sample size, our cohort being almost four times larger size (50 *vs* 221).

While there is a good agreement in the literature about the higher EGFR expression among HPV-negative OPC cases, this is not the case for pEGFR levels. A previous study has reported higher pEGFR levels among HPV-negative OPCs (31), but our results contradict this view. In our series, the strong increase in EGFR levels among HPV-negative OPCs was accompanied by a sensible albeit non-significant decrease in pEGFR levels, to the extent that HPV16-positive samples displayed higher pEGFR positivity than HPV-negative samples (40 *vs* 25%, respectively).

Our rationale to seek a connection between HPV16-*E5* expression and increased EGFR levels stems from the known functional impact of HPV16-E5 on EGFR-mediated signaling. Extracellular binding of the EGF ligand leads to activation of EGFR through intracellular phosphorylation. The activated form pEGFR is internalized, driven to the endosomes and eventually degraded. Presence of HPV16-E5 results in inability for late endosomal acidification (16), preventing pEGFR degradation, which is instead vehiculated again to the cellular surface in activated state, overall resulting in an increased ligand-triggered EGFR response that is maintained even after the EGF stimulus has disappeared (15). Under this view, we initially hypothesized that higher HPV16-*E5* expression would result in increased pEGFR surface recycling, and thus, higher pEGFR positivity. Our results however failed to validate our mechanistic hypothesis, as HPV16-*E5* expression was not related to pEGFR expression in our series. Nevertheless, our results show that HPV16-positive cancers display lower EGFR levels but higher pEGFR/EGFR ratios than HPV-negative cancers. We are not aware of any viral mechanism to explain this puzzling observation.

High EGFR levels are associated with worse prognosis in HNSCC (32), as well as in a subset of HPV-related OPCs (7). In line with these results and with other studies (30, 33), we confirm in our cohort an association between EGFR expression and worse prognosis. The combination of both biomarkers, HPV-relatedness and EGFR or pEGFR expression, could be useful to better characterize OPC patients. When different variables (*i.e*., age, tobacco use) were entered into a multivariate Cox proportional hazards model, HPV status and the combination of HPV-relatedness and EGFR or pEGFR expression were predictors of better OS and DFS. This result contradicts however the study by Romanitan and coworkers, where pEGFR was not found to be an optimal prognostic marker for clinical outcome in OPC patients (31). Furthermore, Bossi and coworkers reviewed in a recent article the prognostic and predictive value of EGFR in HNSCC (34), regarding HPV-positive patients they described different trials evaluating the combined effect of HPV status and EGFR expression on prognosis, showing that patients with EGFR-positive/HPV-negative cancer have the poorest outcomes, while the EGFR negative/HPV-positive group showed the best outcome in line with our results. Nevertheless, they suggest that in recent studies including OPC patients, the prognostic role of EGFR expression was related to the association with HPV-negative tumors, and the added value of EGFR analysis seems to be marginal in respect to HPV (34).

Limited preclinical data investigating the combination of radiotherapy and anti-EGFR antibodies are available and clinical data are still controversial for HPV-related patients (35–37). In our series, only four patients with locally advanced HPV16-positive OPC were treated with anti-EGFR therapy concomitant with radiotherapy. So far, no conclusion can thus be drawn about the use of anti-EGFR therapy, HPV-relatedness and HPV16-*E5* expression and EGFR and pEGFR levels in the context in our series. Interestingly, two of the phase III clinical trials investigating the combination of anti-EGFR therapy with radiotherapy compared to the standard treatment with chemo-radiotherapy for HPV-positive oropharyngeal cancer patients, the RTOG 1026 and the DE-ESCALaTE study, have been recently presented. Both studies demonstrated superiority for the standard treatment (cisplatin-radiotherapy) for HPV-related OPC patients (38–40).

Our study has a number of limitations. First, the HPV-related attributable fraction among OPCs in Mediterranean countries is lower than in other World regions, such as the US or Northern Europe. Thus, despite having analyzed a large global number of OPC cases, our cohort included a low number of HPV-related OPC patients. This reduced effective sample size limits our power to establish the validity of *E5* mRNA expression as a potential biomarker. Second, the analyses for this pilot study were performed on FFPE samples. Although such fixation technique is the most common approach to stabilize tissue prior to anatomopathological analyses, this aggressive chemical treatment may impact the quantity and quality of the retrieved nucleic acids, potentially rendering lower apparent prevalence values of viral DNA and/or mRNA. Third, pEGFR detection is still not widely implemented in the clinical setting, and the determination here applied was semi-quantitative. All in all, these limitations may have hindered the identification of a putative connection between viral HPV16-*E5* expression and the detection of cellular EGFR/pEGFR. On the other hand, the strength of our study is the epidemiology design: we have applied a robust HPV testing method, performed in a single laboratory, to decrease variability; we have only included oropharyngeal samples and excluded other head and neck sub-sites; and we have focused on samples positive only for one single HPV genotype (HPV16).

In summary, our study demonstrates HPV16-*E5* to be highly expressed on HPV16-positive OPC samples. HPV-negative samples expressed significantly more EGFR, while HPV16-positive samples expressed more pEGFR. The combinations of HPV-relatedness and EGFR or pEGFR protein levels are useful biomarkers for prognosis outcome in OPC patients. Specifically, OPC patients with HPV16-positive and EGFR negative cancers present better OS prognosis than patients with HPV-negative and EGFR positive cancers. Further studies are warranted to better understand the role of HPV16-E5 in cancer etiology and its relationship with EFGR and pEGFR expression.

## Author Contributions

MT, MTo, MA, RM, IB, and LA study concept, study design, and interpretation. MT, MM, XL, JG, MG, RH, TB, and AA data acquisition. ST and SM data analysis. MT, MTo, IB, and LA manuscript preparation. All authors reviewed and validated the final version of the manuscript.

### Conflict of Interest Statement

RM has received personal fees and non-financial support from Merck, and personal fees from AstraZeneca, Merck, Brystol-Myers and MSD. MT has received non-financial support from Merck and Astra Zeneca, and personal fees from Merck, Brystol-Myers and AstraZeneca. Cancer Epidemiology Research Program (LA, MM, SM, ST, MTo, MP) has received sponsorship for grants from Merck and co. None of these funders had any role in the data collection, analysis, interpretation of the results, article writing, and decision to publish. The remaining authors declare that the research was conducted in the absence of any commercial or financial relationships that could be construed as a potential conflict of interest.
