# Supplementary material for: The Use of HPV16-E5, EGFR, and pEGFR as Prognostic Biomarkers for Oropharyngeal Cancer Patients
Source: Front Oncol. 2018 Dec 11;8:589. doi: 10.3389/fonc.2018.00589 (PMC6297752; doi:10.3389/fonc.2018.00589)
Supplement: Supplementary file 1 [file Data_Sheet_1.docx]

**SUPPLEMENTARY MATERIAL:**

**Table S1: Descriptive of the oropharyngeal cancer patients by HPV status.**

| **Characteristics** | **OPC patients**  **(N=222)** | | **HPV status** | | | | |
| --- | --- | --- | --- | --- | --- | --- | --- |
|  |  |  | **HPV16**  **positive** | | **HPV/DNA negative** | | ***P* for difference*** |
|  | **N** | **(%)** | **N** | **(%)** | **N** | **(%)** |  |
| **Age at diagnosis** |  |  |  |  |  |  | 0.337^†^ |
| Mean (SD) | 61.3 | (12.0) | 60.3 | (14.6) | 62.2 | (8.7) |  |
| **Gender** |  |  |  |  |  |  | **0.002** |
| Male | 184 | (82.9) | 36 | (67.9) | 148 | (87.6) |  |
| Female | 38 | (17.1) | 17 | (32.1) | 21 | (12.4) |  |
| **Tobacco use** |  |  |  |  |  |  | Matched |
| Non smoker | 60 | (27.0) | 27 | (50.9) | 33 | (19.5) |  |
| <20 cigarettes/day | 57 | (25.7) | 12 | (22.6) | 45 | (26.6) |  |
| ≥20 cigarettes/day | 105 | (47.3) | 14 | (26.4) | 91 | (53.8) |  |
| **Alcohol consumption (n=221)** |  |  |  |  |  |  | **<0.001** |
| Non drinker | 69 | (31.2) | 29 | (54.7) | 40 | (23.8) |  |
| <100 grams/day | 70 | (31.7) | 21 | (39.6) | 49 | (29.2) |  |
| ≥100 grams/day | 82 | (37.1) | 3 | (5.7) | 79 | (47.0) |  |
| **Site** |  |  |  |  |  |  | **<0.001** |
| Tonsil | 97 | (43.7) | 37 | (69.8) | 60 | (35.5) |  |
| BOT | 45 | (20.3) | 9 | (17.0) | 36 | (21.3) |  |
| Tonsil & BOT | 8 | (3.6) | 0 | (0.0) | 8 | (4.7) |  |
| Other^a^ | 72 | (32.4) | 7 | (13.2) | 65 | (38.5) |  |
| **Stage (7^th^ TNM edition)** |  |  |  |  |  |  | 0.171 |
| I | 11 | (5.0) | 1 | (1.9) | 10 | (5.9) |  |
| II | 28 | (12.6) | 3 | (5.7) | 25 | (14.8) |  |
| III | 56 | (25.2) | 14 | (26.4) | 42 | (24.9) |  |
| IVa | 105 | (47.3) | 32 | (60.4) | 73 | (43.2) |  |
| IVb | 17 | (7.7) | 3 | (5.7) | 14 | (8.3) |  |
| IVc | 5 | (2.2) | 0 | (0.0) | 5 | (3.0) |  |
| **Histology** |  |  |  |  |  |  | **<0.001** |
| SCC NOS / SCC Conventional Non Keratinizing | 65 | (29.3) | 17 | (32.1) | 48 | (28.4) |  |
| SCC Conventional Keratinizing | 127 | (57.2) | 17 | (32.1) | 110 | (65.1) |  |
| SCC Basaloid / Papillary | 27 | (12.2) | 19 | (35.8) | 8 | (4.7) |  |
| Other^b^ | 3 | (1.4) | 0 | (0.0) | 3 | (1.8) |  |
| **Tumor differentiation** |  |  |  |  |  |  | **0.008** |
| Grade 1 | 0 | (0.0) | - | - | - | - |  |
| Grade 2 | 75 | (33.8) | 10 | (18.9) | 65 | (38.5) |  |
| Grade 3 | 147 | (66.2) | 43 | (81.1) | 104 | (61.5) |  |
| **Treatment (n=217)** |  |  |  |  |  |  | 0.065 |
| Symptomatic treatment | 17 | (7.8) | 1 | (1.9) | 16 | (9.8) |  |
| Surgery and/or RC/CT | 58 | (26.7) | 11 | (20.8) | 47 | (28.7) |  |
| RT | 35 | (16.1) | 8 | (15.1) | 27 | (16.5) |  |
| RT-Cisplatin | 36 | (16.6) | 15 | (28.3) | 21 | (12.8) |  |
| RT-antiEGFR | 12 | (5.5) | 4 | (7.5) | 8 | (4.9) |  |
| iCT | 59 | (27.2) | 14 | (26.4) | 45 | (27.4) |  |
| **Total** | **222** | **(100.0)** | **53** | **(100.0)** | **169** | **(100.0)** |  |

*Fisher’s exact test for categorical variables.

^†^Paired *t*-test.

Other^a^ mainly includes Soft palate, Vallecula, Posterior wall and Glosso-tonsillar fold.

Other^b^ includes undifferentiated (n=1) and neuroendocrine (n=2) tumors.

Abbreviations: OPC, Oropharyngeal cancer; HPV, Human Papillomavirus; DNA, Deoxyribonucleic acid; SD, Standard Deviation; BOT, Base of the tongue; RC, Radiotherapy; CT, Chemotherapy; EGFR, Epidermal Growth Factor Receptor; iCT, Induction chemotherapy.

Note: HPV16 positive cases are also positive for p16^INK4a^ and E5. Cases matched by tobacco use (see details in Methods section).

**Table S2. Factors associated to HPV16-*E5*, EGFR and pEGFR positivity stratified by HPV status.**

| **Characteristics** | **Positivity for *E5* among HPV16 positive cases (N=53)*** | **EGFR expression** | | | **pEGFR expression** | | |
| --- | --- | --- | --- | --- | --- | --- | --- |
|  |  | **HPV16 positive (N=53)*** | **HPV/DNA negative**  **(N=168)** | | **HPV16 positive (N=53)*** | **HPV/DNA negative**  **(N=169)** | |
|  | **+/No (%)** | **+/No (%)** | **+/No (%)** | **OR** | **+/No (%)** | **+/No (%)** | **OR** |
| **Age at diagnosis** |  |  |  |  |  |  |  |
| Mean (SD) | 60.9 (15.2) | 62.1 (15.9) | 59.5 (10.3) | 0.97 [0.92-1.02] | 60.2 (13.8) | 61.0 (10.6) | 0.995 [0.95-1.04] |
| **Gender** |  |  |  |  |  |  |  |
| Male | 26/36 (72.2) | 13/36 (36.1) | 101/147 (68.7) | Ref. | 13/36 (36.1) | 34/148 (23.0) | Ref. |
| Female | 15/17 (88.2) | 7/17 (41.2) | 18/21 (85.7) | 1.4e+179** | 8/17 (47.1) | 8/21 (38.1) | 3.31 [0.61-17.95] |
| **Tobacco use** |  |  |  |  |  |  |  |
| Non smoker | 22/27 (81.5) | 12/27 (44.4) | 23/32 (71.9) | Matched | 13/27 (48.1) | 4/33 (12.1) | Matched |
| < 20 cigarettes/day | 11/12 (91.7) | 4/12 (33.3) | 30/45 (66.7) |  | 4/12 (33.3) | 11/45 (24.4) |  |
| ≥ 20 cigarettes/day | 8/14 (57.1) | 4/14 (28.6) | 66/91 (72.5) |  | 4/14 (28.6) | 27/91 (29.7) |  |
| **Alcohol consumption** |  |  |  |  |  |  |  |
| Non drinker | 24/29 (82.8) | 11/29 (37.9) | 26/39 (66.7) | Ref. | 11/29 (37.9) | 6/40 (15.0) | Ref. |
| < 100 grams/day | 14/21 (66.7) | 8/21 (38.1) | 36/49 (73.5) | 2.22 [0.44-11.21] | 9/21 (42.9) | 14/49 (28.6) | 5.16 [0.74-35.88] |
| ≥ 100 grams/day | 3/3 (100) | 1/3 (33.3) | 56/79 (70.9) | 1.17 [0.33-4.12] | 1/3 (33.3) | 22/79 (27.8) | 1.94 [0.35-10.86] |
| **Subsite** |  |  |  |  |  |  |  |
| Tonsil | 29/37 (78.4) | 14/37 (37.8) | 44/59 (74.6) | Ref. | 12/37 (32.4) | 17/60 (28.3) | Ref. |
| BOT | 7/9 (77.8) | 4/9 (44.4) | 21/36 (58.3) | 0.36 [0.09-1.45] | 5/9 (55.6) | 9/36 (25.0) | 0.57 [0.14-2.38] |
| Tonsil & BOT | - | - | 6/8 (75.0) | 0.25 [0.03-2.28] | - | 0/8 (0.0) | -*** |
| Other^a^ | 5/7 (71.4) | 2/7 (28.6) | 48/65 (73.8) | 0.45 [0.11-1.84] | 4/7 (57.1) | 16/65 (24.6) | 0.33 [0.08-1.35] |
| **Stage (7th TNM edition)** |  |  |  |  |  |  |  |
| I | 1/1 (100) | 0/1 (0.0) | 7/10 (70.0) | Ref. | 0/1 (0.0) | 0/10 (0.0) | -*** |
| II | 3/3 (100) | 2/3 (66.7) | 19/25 (76.0) | 2.82 [0.30-26.94] | 2/3 (66.7) | 3/25 (12.0) | Ref.^‡^ |
| III | 11/14 (78.6) | 3/14 (21.4) | 29/42 (69.0) | 1.50 [0.16-13.92] | 7/14 (50.0) | 14/42 (33.3) | 2.73 [0.28-26.96] |
| IVa | 23/32 (71.9) | 15/32 (46.9) | 50/72 (69.4) | 1.31 [0.16-10.76] | 11/32 (34.4) | 16/73 (21.9) | 2.73 [0.28-26.96] |
| IVb | 3/3 (100) | 0/3 (0.0) | 10/14 (71.4) | 1.73 [0.15-19.97] | 1/3 (33.3) | 6/14 (42.9) | -*** |
| IVc | - | - | 4/5 (80.0) | 1.37 [0.07-25.91] | - | 3/5 (60.0) | -*** |
| **Histology** |  |  |  |  |  |  |  |
| SCC NOS / SCC Conventional non keratinizing | 13/17 (76.5) | 8/17 (47.1) | 25/48 (52.1) | Ref.^†^ | 8/17 (47.1) | 11/48 (22.9) | Ref. |
| SCC Conventional keratinizing | 13/17 (76.5) | 6/17 (35.3) | 89/110 (80.9) | **4.81 [1.45-15.93]** | 8/17 (47.1) | 29/110(26.4) | 1.50 [0.55-4.07] |
| SCC basaloid/papillary | 15/19 (78.9) | 6/19 (31.6) | 4/8 (50.0) | 0.81 [0.06-10.94] | 5/19 (26.3) | 1/8 (12.5) | 0.43 [0.03-5.51] |
| Other^b^ | - | - | 1/2 (50.0) | -*** | - | 1/3 (33.3) | -*** |
| **Tumor differentiation** |  |  |  |  |  |  |  |
| Grade 1 | - | - | - |  | - | - | - |
| Grade 2 | 8/10 (80.0) | 4/10 (40.0) | 52/65 (80.0) | Ref.^†^ | 4/10 (40.0) | 16/65 (24.6) | Ref. |
| Grade 3 | 33/43 (76.7) | 16/43 (37.2) | 67/103 (65.0) | **0.23 [0.06-0.84]** | 17/43 (39.5) | 26/104 (25.0) | 0.64 [0.25-1.63] |
| **Treatment** |  |  |  |  |  |  |  |
| Symptomatic treatment | 1/1 (100) | 0/1 (0.0) | 13/16 (81.3) | Ref. | 0/1 (0.0) | 6/16 (37.5) | Ref. |
| Surgery and/or RT/CT | 10/11 (90.9) | 1/11 (9.1) | 34/47 (72.3) | 1.09 [0.17-6.83] | 1/11 (9.1) | 5/47 (10.6) | 0.41 [0.07-2.39] |
| RT | 6/8 (75.0) | 6/8 (75.0) | 18/27 (66.7) | 0.22 [0.03-1.84] | 6/8 (75.0) | 7/27 (25.9) | 0.61 [0.10-3.61] |
| RT-Cisplatin | 12/15 (80.0) | 6/15 (40.0) | 18/21 (85.7) | 2.46 [0.28-21.61] | 6/15 (40.0) | 9/21 (42.9) | 3.37 [0.28-40.12] |
| RT-anti-EGFR | 2/4 (50.0) | 1/4 (25.0) | 6/8 (75.0) | 0.63 [0.03-12.62] | 1/4 (25.0) | 2/8 (25.0) | 1.41 [0.06-34.84] |
| iCT | 10/14 (71.4) | 6/14 (42.9) | 26/44 (59.1) | 0.30 [0.03-2.82] | 7/14 (50.0) | 13/45 (28.9) | 0.77 [0.10-6.17] |
| **TOTAL** | **41/53 (77.4)** | **20/53 (37.7)** | **119/168 (70.8)** |  | **21/53 (39.6)** | **42/169 (24.9)** |  |

*, OR cannot be calculated because the outcome does not vary in any group; **, convergence not achieved; -***, omitted because of no within-group variance; ^†^*P*<0.05; ^‡^*P*<0.01.

Other^a^ mainly includes Soft palate, Vallecula, Posterior wall and Glosso-tonsillar fold; Other^b^ includes undifferentiated (n=1) and neuroendocrine (n=2) tumors.

Abbreviations: +, number of positive cases; No, Total number of cases; OR, Crude Odds Ratio; SD, Standard Deviation; Ref., Reference; INF: Infinite; HPV: Human Papillomavirus; EGFR: Epidermal Growth Factor Receptor; BOT: Base of the tongue; SCC: Squamous cell carcinoma; NOS: Not otherwise specified; RT: Radiotherapy; CT: Chemotherapy; iCT: Induction chemotherapy.

Note: Total numbers might not sum due to missing values. HPV16 positive cases are also positive for p16^INK4a^. Cases matched by tobacco use (see details in Methods section).
